# Pd immobilization biguanidine modified Zr-UiO-66 MOF as a reusable heterogeneous catalyst in Suzuki–Miyaura coupling

**DOI:** 10.1038/s41598-021-00991-3

**Published:** 2021-11-08

**Authors:** Hojat Veisi, Mozhdeh Abrifam, Sheida Ahany Kamangar, Mozhgan Pirhayati, Shokoufeh Ghahri Saremi, Mohammad Noroozi, Taiebeh Tamoradi, Bikash Karmakar

**Affiliations:** 1 Department of Chemistry, Payame Noor University, Tehran, Iran; 2 Department of Applied Chemistry, Faculty of Science, Malayer University, Malayer, Iran; 3 Center for Research and Development of Petroleum Technologies at Kermanshah, Research Institute of Petroleum Industry (RIPI), Tehran, Iran; 4 Department of Chemistry, Production Technology Research Institute-ACECR, Ahvaz, Iran; 5 Department of Chemistry, Gobardanga Hindu College, 24-Parganas (North), Gobardanga, India

**Keywords:** Catalysis, Inorganic chemistry

## Abstract

In recent days, nanohybrid metal organic frameworks (MOF) have been considered as next generation catalysts due to their unique features like large surface to volume ratio, tailorable geometry, uniform pore sizes and homogeneous distribution of active sites. In this report, we address the biguanidine modified 3D Zr-centred MOF UiO-66-NH_2_ following a post synthetic modification approach. Utilizing the excellent chelating ability of biguanidine, Pd ions are immobilized over the host matrix MOF. The as-synthesized material was physicochemically characterized using a broad range of analytical techniques like FT-IR, electron microscopy, EDS, elemental mapping, XRD and ICP-OES. Subsequently the material has been catalytically employed in the classical Suzuki–Miyaura coupling towards the synthesis of diverse biphenyl derivatives at sustainable conditions. There are very few reports on the covalently modified MOFs towards the organic coupling reactions. The catalyst has been isolated by centrifugation and recycled in 9 consecutive runs with almost insignificant leaching and minute decrease in reactivity.

## Introduction

In the recent past, scientists have witnessed unprecedented progress in catalysis, particularly after the inception and involvement of designed and engineered nanomaterials, in view of economic and environmental reasons^1–3^. With time heterogeneous catalysts have been further bejeweled when MOFs came into prominence. MOFs are porous crystalline coordination polymers (PCCP) with well-defined pore surfaces having metal nodes, being connected to organic ligands like aromatic polycarboxylates or nitrogenous heterocycles^4,5^. They acquire exceptionally large surface to volume ratio, adjustable pore dimensions, defined crystal environment, homogeneously dispersed catalytic sites throughout the matrix, tunable metal concentration and accessible to varied chemical modifications^7–10^. One of the key features of MOFs is the opportunity to architect them by selecting suitable ligand of proper size and geometry, different type of metal nodes with variable coordinating fashions that makes the pore dimensions, geometry and rigidity of the corresponding MOF absolutely predictable^11,12^. Due to such kind of exclusive features and advantages, MOF derivatives have been widely applied in gas adsorption, storage and separation, luminescence, water treatment, sensing, proton conductivity, magnetics, energy related applications, drug delivery specifically in cancer therapy, and as nano-reactor in heterogeneous catalysis^13–17^. However, questions have been raised regarding the limitation in MOFs, particularly about their physical and thermal stability^6^. In consequences to that, dealing with heterogeneous catalytic support, we selected UiO-66-NH_2_, a Zr-terephthalate derived MOF, which bears significant chemical, thermal and mechanical stability as well as suitable towards post-functionalizations. The UiO-66 skeleton is constructed with [Zr_6_O_4_(OH_)4_] octahedral secondary building units (SBU) and 1,4-benzene dicarboxylate (BDC) derivatives. In bond connectivity, Zr_6_ cluster is coordinated to BDC in 1:12 unit ratio three dimensionally thus affording a hierarchical framework^18–21^. There are several reports on the synthesis and catalytic applications of various MOF derivatives being synthesized from pre-modified ligands and post-immobilized metal ions or metal nanoparticles thereon^22–29^. However, synthetic organic applications of active metal adorned post-functionalized MOFs, has not been much explored and there are ample scopes to develop this area^30–34^. This has persuaded us to design the Zr-UiO-66 MOF using 2-amino-1,4-dicarboxylic acid ligand and subsequently covalent functionalization with cyano guanidine to generate a biguanidine moiety in situ. Biguanidine is a recognized and excellent chelating ligand and we exploited it to anchor Pd ions at the outer-shell of Zr-UiO-66-NH_2_.

**Figure 1:**
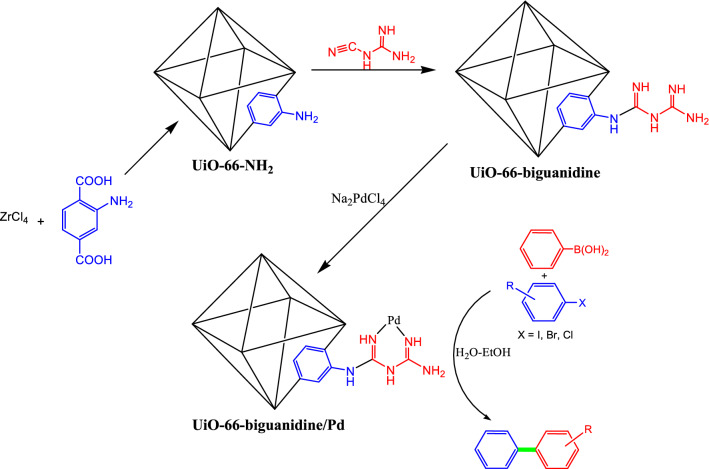
Sequential synthesis of UiO-66-biguanidine/Pd nanocomposite catalyst towards the Suzuki–Miyaura coupling reactions.

In synthetic organic chemistry carbon–carbon bond formation is measured as one of the most fundamental and challenging reactions^35–37^. Among the several such categories, Suzuki–Miyaura coupling is considered as a protagonist and used in the synthesis of diverse symmetric and asymmetric biphenyl compounds which have otherwise applications as important hypertensive, antimicrobial, fungicide, anti-diabetic and analgesic drugs^38–43^. There are prolific reports on the Suzuki–Miyaura coupling methodology over different Pd catalysts^44–49^. However, the Pd functionalized biguanidine modified Zr-UiO-66 MOF (Fig. 1) on the Suzuki coupling is not reported so far. Hence, we introduce a green and competent protocol on the Suzuki reaction by coupling a wide range of aryl halides with phenylboronic acid over UiO-66-biguanidine/Pd nanocomposite. Operational simplicity, green reaction conditions, simple and inexpensive procedure, high efficiency, short reaction times, easy separation of the catalyst and reusability for several consecutive cycles are the key advantages of this protocol.

## Experimental

### Preparation of UiO-66-biguanidine

UiO-66-biguanidine is synthesized as our previous report^50^.

### Preparation of UiO-66-biguanidine/Pd

A uniformly dispersed solution of UiO-66-biguanidine was prepared by sonication in 50 mL H_2_O which was followed by addition an aqueous solution of Na_2_PdCl_4_ (15 mL, 2 mg mL^−1^) as Pd precursor and stirred for 12 h. The resulting solid was collected by centrifuge, washed twice with 20 mL DI water and then with 20 mL ethanol. Finally, the UiO-66-biguanidine/Pd was dried in vacuum at 60 °C for 24 h. The Pd load in the material was found 0.18 mmol/g, being estimated via ICP-OES method.

### Suzuki–Miyaura coupling over UiO-66-biguanidine/Pd nanocomposite catalyst

A mixture of Aryl halide, phenyl boronic acid and K_2_CO_3_ in 1:1:2 molar ratio was stirred in aqueous EtOH (3 mL) and then the catalyst (30 mg, 0.1 mol %) was added and gently warmed at 50 °C for proposed time. After completion (by TLC), EtOAc was added to the mixture and the catalyst was isolated off. The entire mixture was soaked over anhydrous Na_2_SO_4_ and the organic layer obtained was evaporated to get the biphenyl product in almost pure form. They were further purified by passing over a silica gel (100–200 mesh) filter column with 5% EtOAc/Hexane as eluent.

## Results and discussions

### Catalyst characterization data analysis

**Figure 2:**
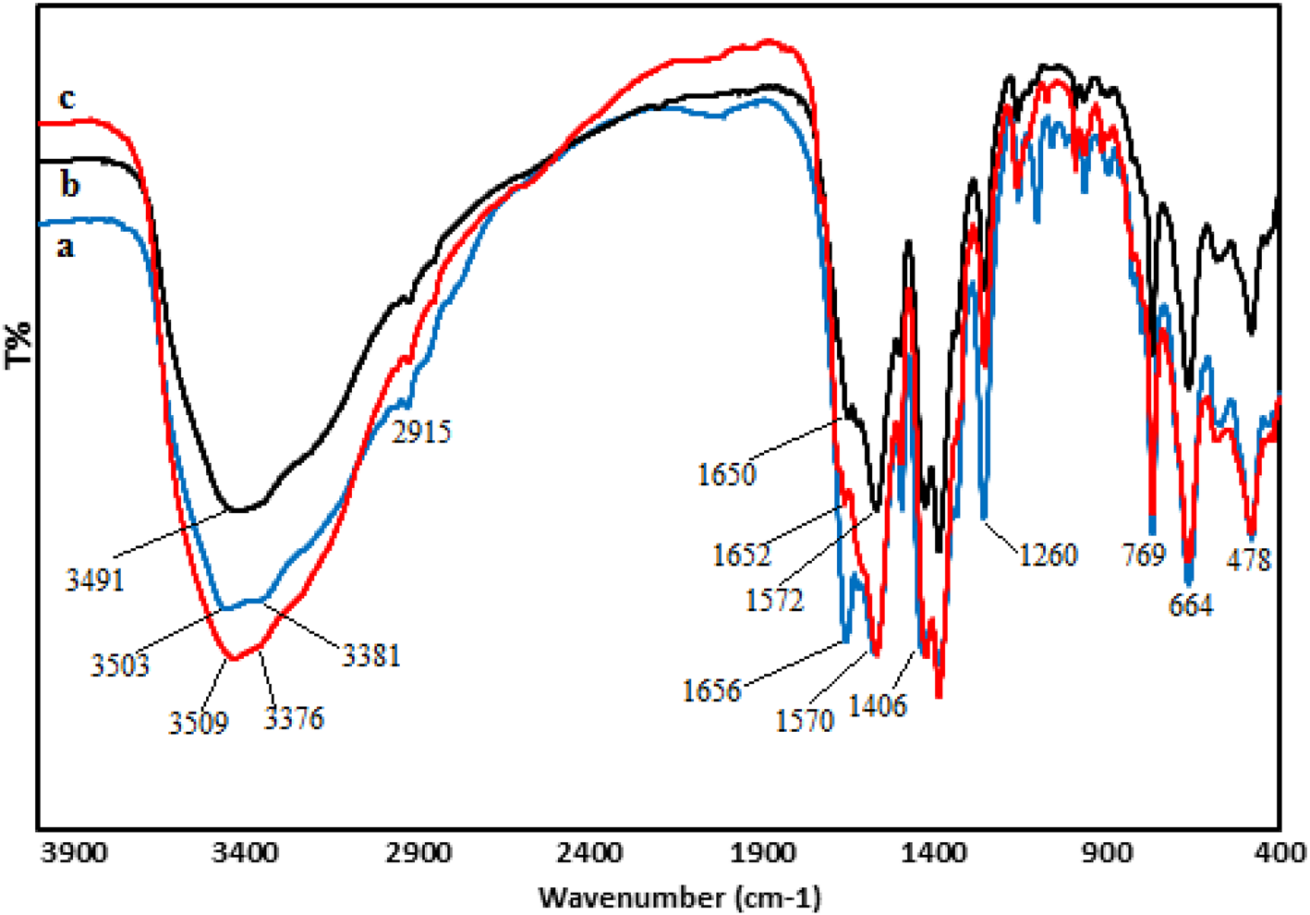
FT-IR spectra of UiO-66-NH_2_ (**a**), UiO-66-biguanidine (**b**) and UiO-66-biguanidine/Pd (**c**).

Physicochemical characteristics of the as synthesized catalyst were determined following a detailed analysis over FT-IR, SEM, EDX, elemental mapping, TEM and XRD study. A comparative FT-IR analysis between UiO-66-NH_2_, UiO-66-biguanidine and UiO-66-biguanidine/Pd materials have been showed in Fig. 2. The bare UiO-66-NH_2_ can be identified by the characteristic vibrations at 1406 and 1570 cm^−1^, attributed to the symmetric and asymmetric stretching of COOH groups from BDC (Fig. 2a). Amino function can be detected by the H–N–H scissoring, C-NH_2_ stretching, N–H symmetric and asymmetric stretching vibrations at 1260 cm^−1^, 1656 cm^−1^, 3381 cm^−1^ and 3503 cm^−1^ respectively^20,26^. In the synthesis of UiO-66-biguanidine, the UiO-66-NH_2_ reacts with cyano guanidine and the change in bonding can be evidenced from FT-IR spectrum. In Fig. 2b, representing the UiO-66-biguanidine, the two N–H stretching peaks are disappeared while the other peaks remained almost unaltered, as the free amine is replaced by guanidine scaffold^50^. Though, no considerable differences being detected between the spectrum of Fig. 2b from Fig. 2c, except a sight shifting of peaks at lower region due to the strong coordination of Pd nanoparticles to the biguanidine ligand (Fig. 2c).

**Figure 3:**
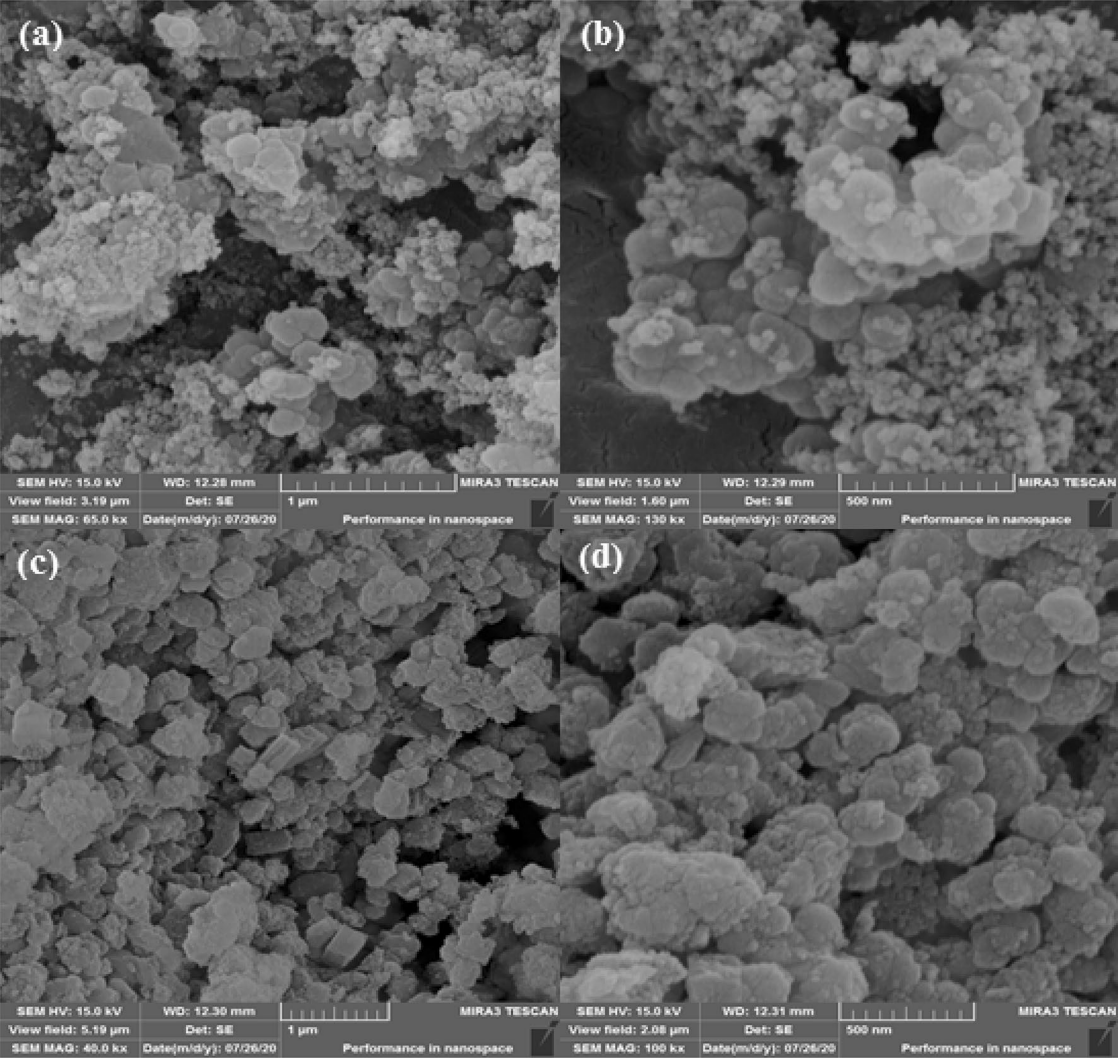
SEM images of UiO-66-NH_2_ (**a**,**b**); and UiO-66-biguanidine/Pd nanocomposite (**c**,**d**).

The particle size, shape, dimensions and textural morphology was investigated over SEM. Figure 3 depicts the particle images at different magnifications. The nanocrystals are homomorphic and cubical in shape with an average dimension of 40 to 80 nm. The Pd association or surface functionalization leaves no significant changes in the apparent morphology. The particles seem to be somewhat agglomerated due to high concentration during sampling.

**Figure 4:**
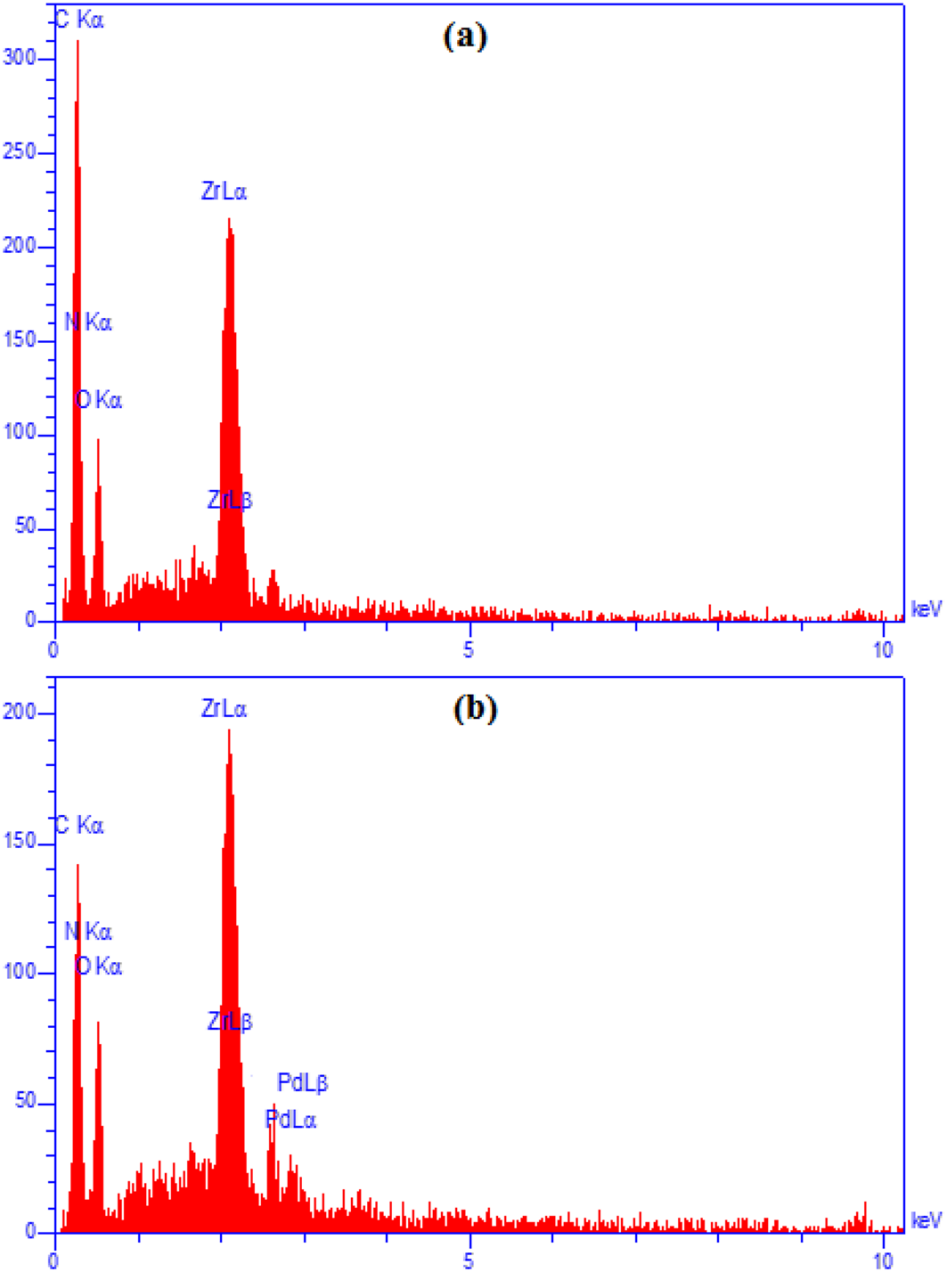
EDX spectra of UiO-66-NH_2_ (**a**); and UiO-66-biguanidine/Pd nanocomposite (**b**).

The elemental composition of UiO-66-NH_2_ and the final material was ascertained by EDX analysis as shown in Fig. 4. The profile of UiO-66-NH_2_ displays the signals of Zr, N and O atoms, indicating Zr and N, O species being contributed from as SBU and BDC respectively (Fig. 4a). Figure 4b demonstrates the profile of UiO-66-biguanidine/Pd, presenting the same elements along with Pd and C. C species correspond to the attached organic ligand (biguanidine) and Pd being immobilized over it.

**Figure 5:**
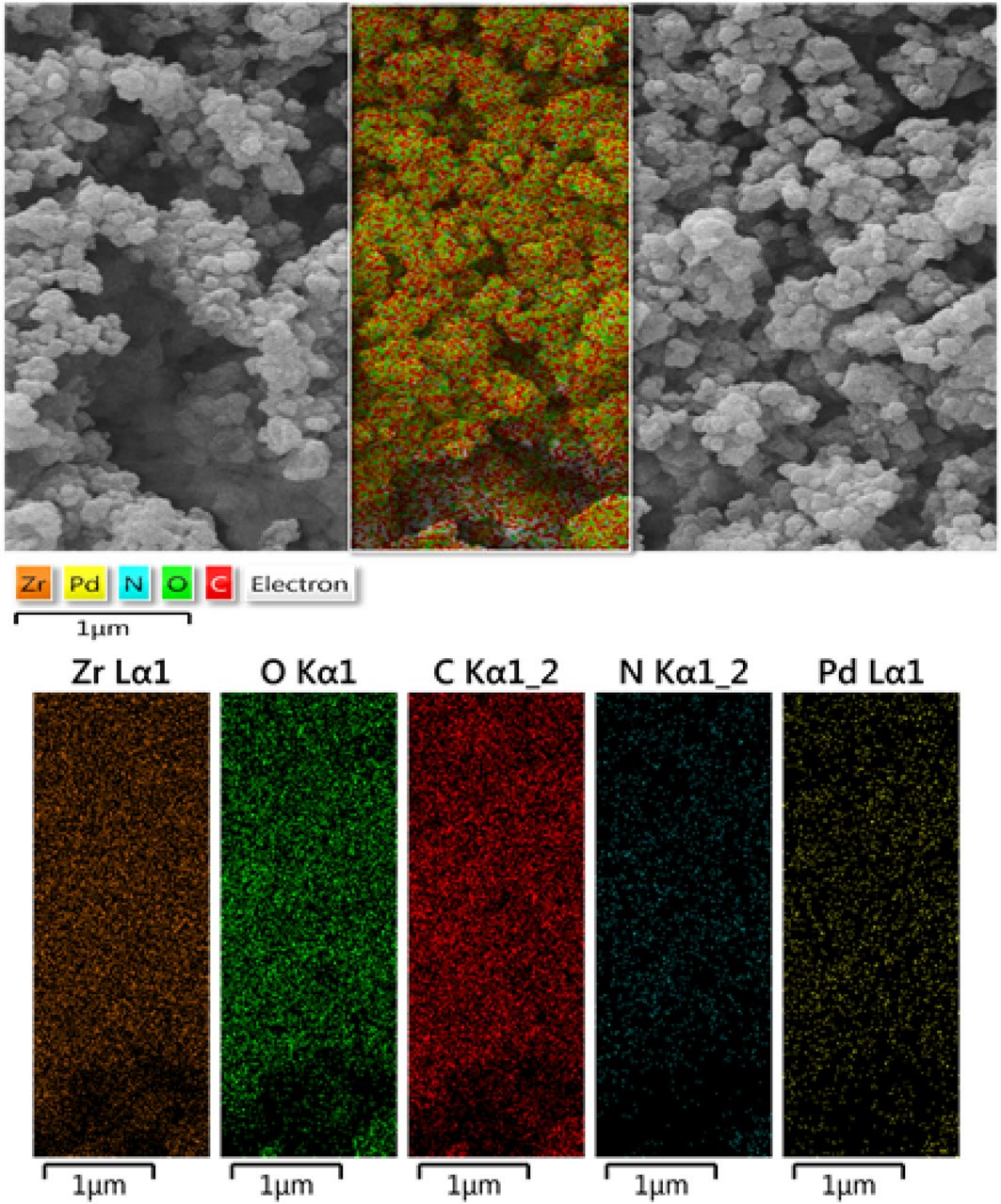
Elemental mapping of nanocomposite UiO-66-biguanidine/Pd with the atomic distribution of Zr, O, C, N, and Pd.

Additionally, surface allocation of the corresponding elements in UiO-66-biguanidine/Pd was ascertained by the X-ray elemental mapping study. A segment of the SEM image of the catalyst was scanned by X-ray and the outcome is displayed in Fig. 5. It evidently shows the homogeneous dispersion of Zr, C, O, N and Pd*.* Obviously, Zr is having much higher density than Pd as the former is the basic constructive unit of the material. Again, the dispersed Pd is observed to be of higher concentration than N. This can be corroborated as Pd is not only bonded to the guanidine moiety, but also associated inside the MOF structure. Nevertheless, the uniform distribution of Pd has significant importance behind its outstanding catalytic activity.

**Figure 6:**
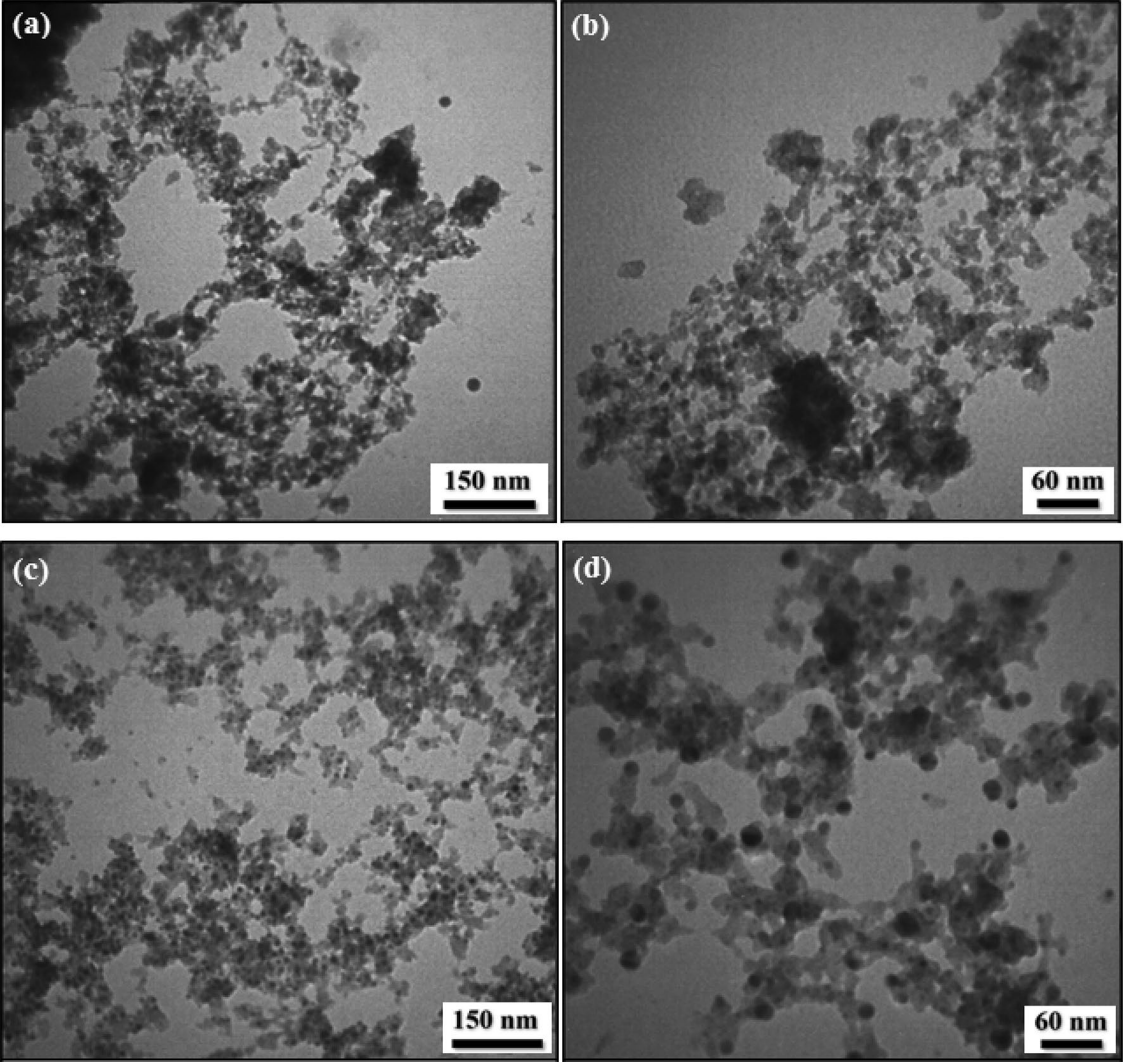
TEM images of UiO-66-NH_2_ (**a**,**b**); and UiO-66-biguanidine/Pd nanocomposite (**c**,**d**).

The TEM images of UiO-66-NH_2_ and UiO-66-biguanidine/Pd materials are presented in Fig. 6. As the Fig. 6a,b shows, UiO-66-NH_2_ represent a poorly crystalline discrete structure. However, in the final material the Pd NPs are clearly can be observed as round shaped black dots spread over the UiO-66-biguanidine MOF support. The monodispersed NPs have an average diameter of 20 nm (Fig. 6c,d).

**Figure 7:**
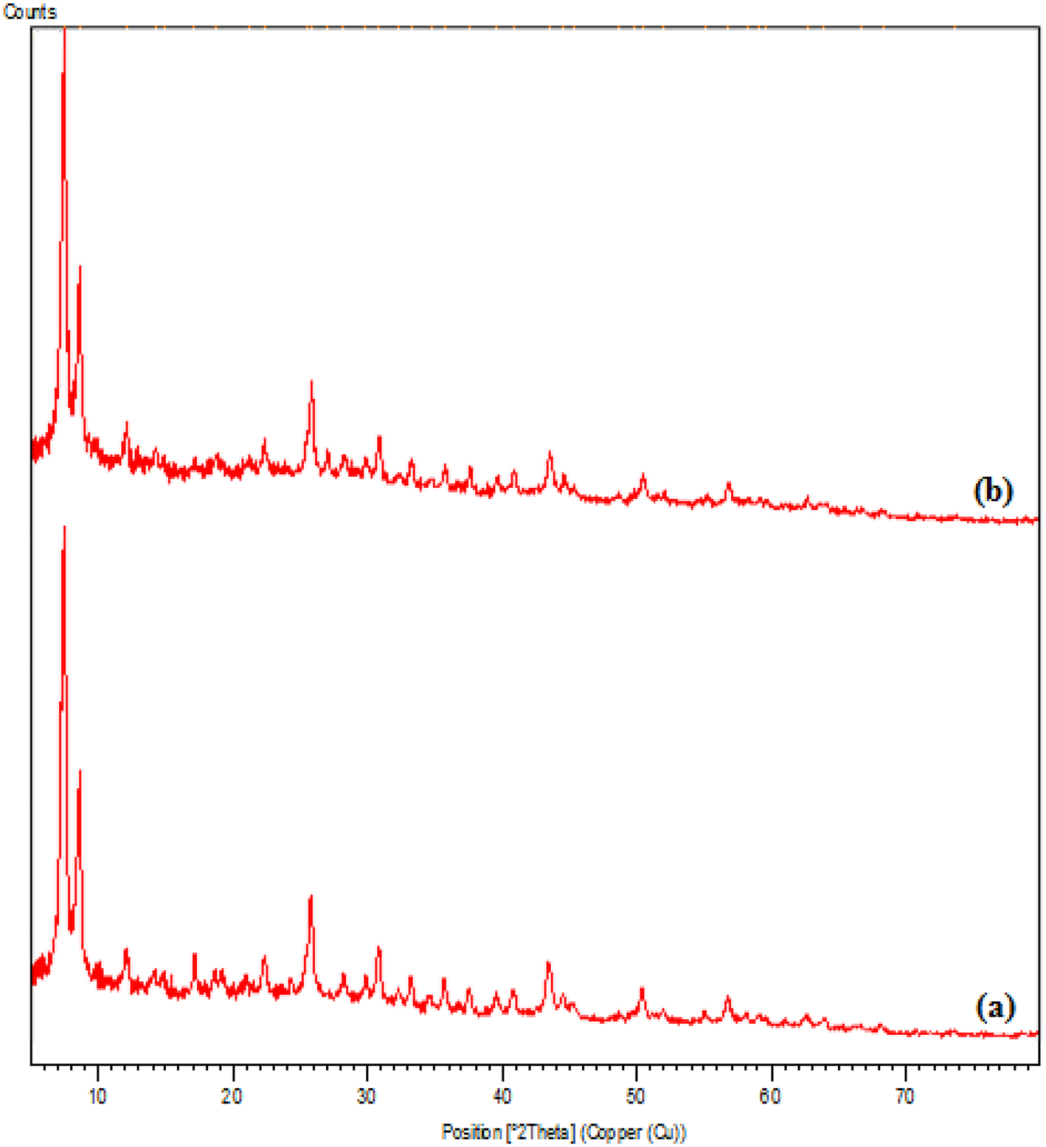
XRD patterns of UiO-66-NH_2_ (**a**); and UiO-66-biguanidine/Pd (**b**).

XRD study were carried out to investigate the phase and crystalline morphology of UiO-66-NH_2_ and UiO-66-biguanidine/Pd, being depicted in Fig. 7. Evidently, both the materials have poor crystallinity, as predicted from TEM image. XRD profile of UiO-66-NH_2_ represents three characteristic diffraction peaks appeared at 2θ = 7.3, 8.7 and 26.1° respectively, being comparable to previous literature^51^. Apparently, both the materials exhibit roughly similar XRD patterns, signifying conservation of internal framework upon post-synthetic modifications. However, no significant peaks were detected due to the attached Pd species.

**Figure 8:**
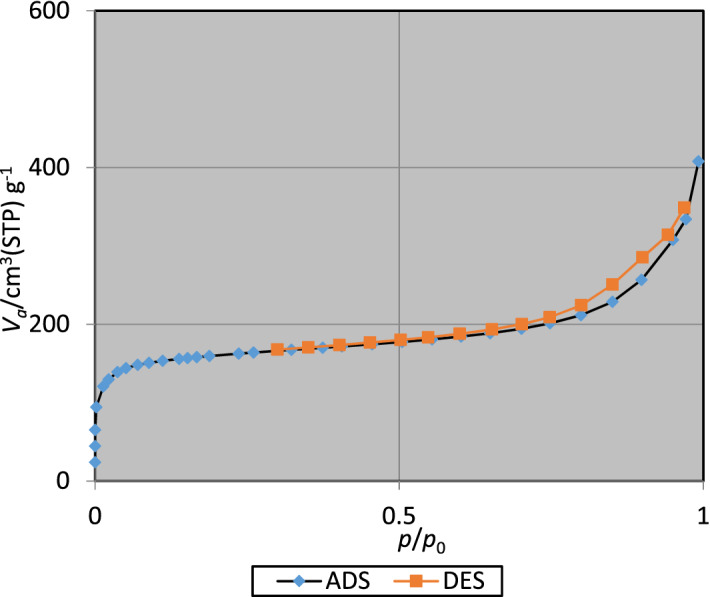
N_2_-adsorption isotherms of UiO-66-biguanidine/Pd.

Surface analysis of UiO-66-biguanidine and UiO-66-biguanidine/Pd materials were investigated through nitrogen adsorption–desorption study and the corresponding outcomes have been presented in Table 1. Langmuir surface area of the two materials were found 831 and 629 m^2^/g respectively. The lower surface area of the latter can be anticipated due to immobilization of Pd nanoparticles and partially blocking the pores lying on the surface of MOF. This is the reason for its reduced pore volume and pore diameter as compared to its precursor. Langmuir isotherm of UiO-66-biguanidine/Pd has been shown in Fig. 8, which is a typical type I isotherm indicating the material to be microporous in nature.

**Table 1:** Nitrogen adsorption–desorption data for UiO-66-biguanidine and UiO-66-biguanidine/Pd.

Entry	Samples	S_BET_ (m^2^g^−1^)	Total pore volume (cm^3^g^−1^)	Mean pore diameter (nm)
1	UiO-66-biguanidine	831	0.52	0.58
2	UiO-66-biguanidine/Pd	629	0.41	0.51

On having scrupulous catalytic characterizations and analyses, it was the time for catalytic explorations and the effect was studied in Suzuki–Miyaura reaction following a straightforward pathway. On completion, the catalyst was isolated by centrifuge for further runs. Nevertheless, with the aim of having the standardized conditions, a probe reaction between phenyl boronic acid and 4-boromotoluene was set to notice the effect of variable conditions like solvent, applied base, Pd load and temperature and the results are documented in Table 2. The investigations were started with 0.1 mol% Pd loaded catalyst and K_2_CO_3_ as base (2.0 mmol). When we investigated the probe in diverse solvents like DMF, toluene, EtOH and H_2_O, the optimum result was obtained in aqueous EtOH (1:1) (Table 3, entries 1–5). On the other hand, while carrying out the reaction in the absence of base, a weak productivity was encountered, implying its significance (Table 4, entry 4). Thereby, we investigated the effect of various bases like Et_3_N, Na_2_CO_3_ and K_2_CO_3_, when the last one afforded the highest yield (Table 4, entries 1–3). Among the different Pd loaded catalysts, we found the 0.1 mol% worked as the optimum (Table 5, entries 1–3). There was no product at all over the Pd free bare catalyst (Table 5, entry 4). Finally, we also studied the effect of temperature keeping the best solvent, catalyst and base. From Table 6 it is evident that the reaction does not produce satisfactory yields at lower temperatures like 25 °C and 40 °C (entry 2,4) and optimum result was obtained at 50 °C (entry 1).

**Table 2:**
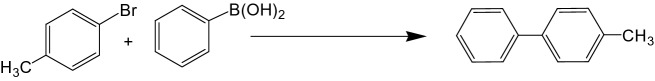
The optimization study in the reaction of 4-bromotoluene with phenyl boronic acid over UiO-66-biguanidine/Pd^a^.

**Table 3:** Screening of solvent.

Entry	Solvent	Time (Min)	Yield (%)
1	DMF	60	65
2	Toluene	60	55
3	EtOH	60	70
4	H_2_O	120	50
**5**	**EtOH/H**_**2**_**O (1:1)**	**20**	**96**

Reaction conditions: 4-methylbromobenzene (1.0 mmol), phenylboronic acid (1.0 mmol), UiO-66-biguanidine/Pd, K_2_CO_3_ as base (2 mmol) and solvent (3 mL) at 50 °C; Isolated yield.

**Table 4:** Screening of base.

Entry	Base	Time (Min)	Yield (%)
**1**	**K**_**2**_**CO**_**3**_	**20**	**96**
2	Et_3_N	60	60
3	Na_2_CO_3_	60	70
4	No base	120	Trace

Reaction conditions: 4-methylbromobenzene (1.0 mmol), phenylboronic acid (1.0 mmol), UiO-66-biguanidine/Pd, EtOH/H_2_O (1:1) as solvent (3 mL) at 50 °C; Isolated yield.

**Table 5:** Variation of catalyst load.

Entry	Pd (mol%)	Time (Min)	Yield (%)
**1**	**0.1**	**20**	**96**
2	0.05	30	70
3	0.2	20	96
4	0.0	120	0

Reaction conditions: 4-methylbromobenzene (1.0 mmol), phenylboronic acid (1.0 mmol), K_2_CO_3_ as base (2 mmol), EtOH/H_2_O (1:1) as solvent (3 mL) at 50 °C; Isolated yield.

**Table 6:** Variation of temperature.

Entry	T (°C)	Time (Min)	Yield (%)
**1**	**50**	**20**	**96**
2	25	120	75
3	60	20	96
4	40	30	70

Reaction conditions: 4-methylbromobenzene (1.0 mmol), phenylboronic acid (1.0 mmol), UiO-66-biguanidine/Pd (0.1 mol% Pd), K_2_CO_3_ as base (2 mmol), EtOH/H_2_O (1:1) as solvent (3 mL); Isolated yield.

After having the optimized conditions, we wished to investigate their generalizations and scope. Diverse array of biaryls were synthesized by coupling various haloarenes and phenylboronic acid following the stabilized conditions (Table 7). In the entire scenario, the chlorobenzenes were found to react sluggishly as compared to bromo or iodoarenes which is manifested from their yields and reaction times. A wide variety of bromo and iodoarenes having electron withdrawing (COCH_3_) or electron donating substituent (CH_3_, OCH_3_, NH_2_, OH) were found very well compatible under the optimized conditions. Markedly, we achieved very good productivity with heteroaryl halides like 2-bromothiophene and 2-iodothiophene as substrate (Table 7, entries 16–17) coupled fruitfully with high yields. All the reactions were completed within 10–60 min except those with chloroarenes which were mostly sluggish. This can be explained based on strong electronegativity and the poor leaving capacity of Cl atom.

**Table 7:** Catalytic activity of UiO-66-biguanidine/Pd nanocomposite in Suzuki–Miyaura coupling reactions.


Entry	RC_6_H_4_X	X	Time (min)	Yield (%)^b^
1	H	I	10	98
2	H	Br	15	98
3	H	Cl	120	50
4	4-CH_3_	I	10	96
5	4-CH_3_	Br	20	96
6	4-CH_3_	Cl	120	45
7	4-COCH_3_	I	20	96
8	4-COCH_3_	Br	45	96
9	4-COCH_3_	Cl	120	40
10	4-CH_3_O	I	30	96
11	4-CH_3_O	Br	45	90
12	4-NH_2_	I	45	90
13	4-NH_2_	Br	90	82
14	4-OH	I	60	90
15	4-OH	Br	120	85
16	2-Thienyl	I	60	92
17	2-Thienyl	Br	120	88

^a^Reaction conditions: 1.0 mmol arylhalide,1.0 mmol phenylboronic acid, 2 mmol K_2_CO_3_, 0.03 g catalyst (0.1 mol% Pd), 3 mL of H_2_O/EtOH (1:1), 50 °C, ^b^Isolated yield.

### Reusability study of catalyst

**Figure 9:**
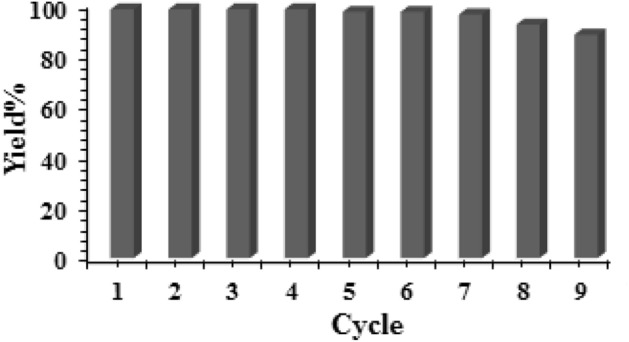
The recycling of the UiO-66-biguanidine/Pd nanocomposite.

**Figure 10:**
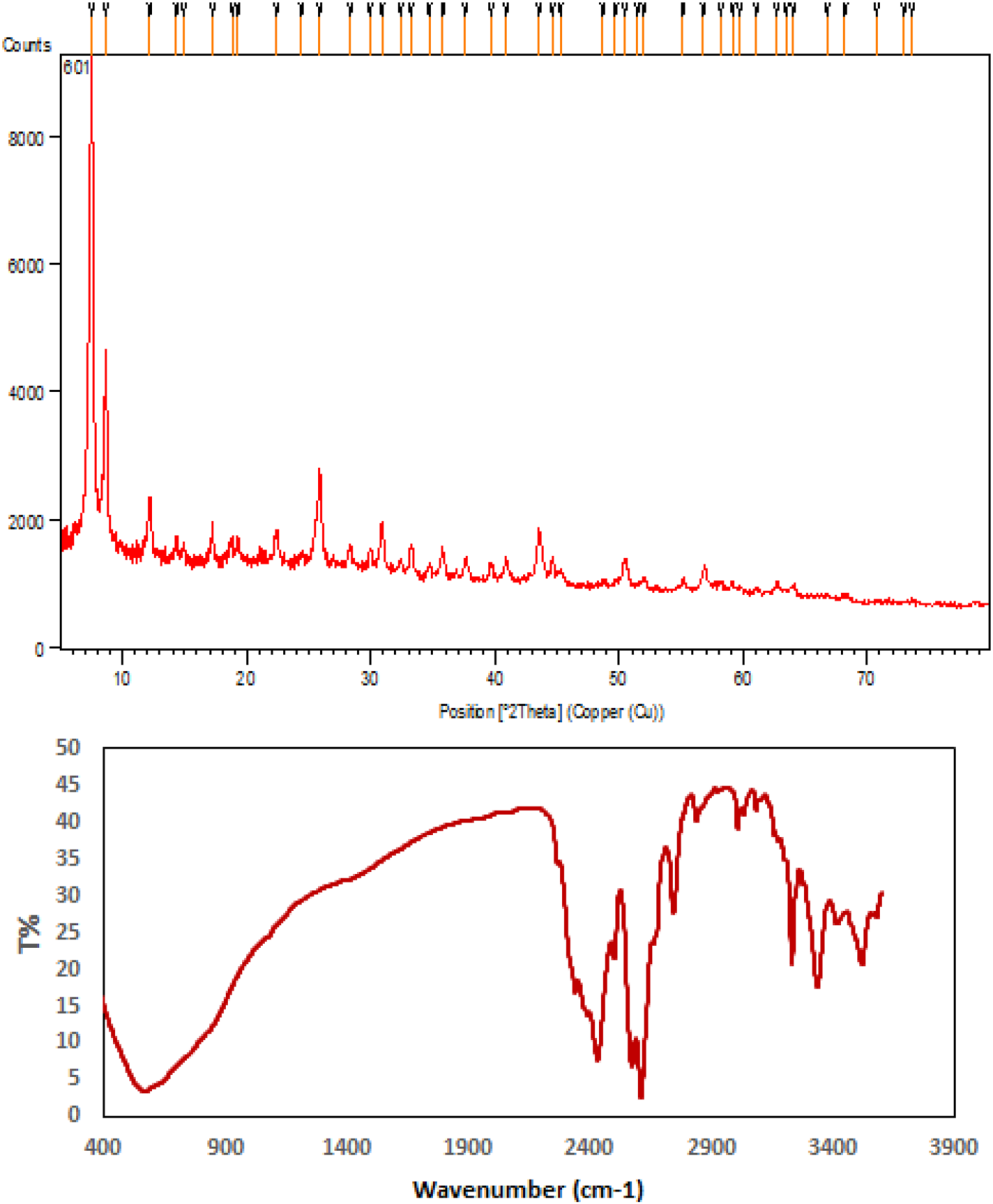
XRD and FT-IR data for reused UiO-66-biguanidine/Pd catalyst after 9 runs.

In consideration of heterogeneous green catalysis, an exploration of recyclability of the corresponding catalyst seems a crucial principle. On completion of a fresh batch of probe, it was isolated by centrifuge and rinsed thoroughly with aqueous EtOH. Subsequently, it was dried and reused in the further cycles. The UiO-66-biguanidine/Pd nanocomposite displayed significant activity up to 7 successive cycles without considerable reduction in reactivity. However, the yield fell down to 90% and 86% in 8th and 9th cycle (Fig. 9). This could be due to aerial oxidation of active species, agglomeration of Pd NPs or deposition of organic species over them. We further analyzed the structural morphology of UiO-66-biguanidine/Pd catalyst after recycling 9 times by using XRD and FT-IR. The results shown that the catalyst retains its initial morphology and structure without any change (Fig. 10), which in turn validates the robustness of our material.

### Uniqueness of our results

To ascertain the distinctiveness of our devised catalytic system, a systematic comparison with a number of other protocols in the coupling between phenylboronic acid and bromo and iodooarenes has been done. Evidently, the UiO-66-biguanidine/Pd nanocomposite reveals a superior result in terms of TOF, as shown in Table 8.

**Table 8:** Catalytic Comparison in the reaction between phenyl boronic acid and iodobenzene.

Entry	Catalyst (mol%)	Conditions	X	TOF (h^−1^)^a^	Refs
1	Bis(oxamato)palladate(II) complex (5)	Et_3_N, *n*‐Bu_4_NBr, 120 °C	I, Br	7.8, 6.5	^52^
2	NHC‐Pd(II) complex (0.2)	K_3_PO_4_.3H_2_O, H_2_O, TBAB, 40 °C	I, Br	98, 75	^53^
3	SiO_2_‐pA‐Cyan‐Cys‐Pd (0.5)	K_2_CO_3_, H_2_O, 100 °C	I, Br	38, 32	^54^
4	Pd_3_(dba) (1)	K_3_PO_4_, THF, 80 °C	Br	3.2	^55^
5	Pd–BOX (2)	K_2_CO_3_, DMF, 70 °C	I	8.3	^56^
6	γ ‐Fe_2_O_3_‐acetamidine‐Pd (0.12)	Et_3_N, DMF, 100 °C	I, Br	1600, 1600	^57^
7	Pd‐isatin Schiff base‐γ‐Fe_2_O_3_ (0.5, 1.5)	Et_3_N, Solvent‐free, 100 °C	I, Br	380, 85.7	^58^
8	UiO-66-biguanidine/Pd (0.1)	K_2_CO_3,_ H_2_O-EtOH_,_ 50 °C	I, Br	11,870, 3920	this work

^a^TOF, turnover frequencies (TOF = (Yield/Time)/Amount of catalyst (mol).

## Conclusion

In summary, we introduce a biguanidine modified Zr-UiO-66 metal organic framework with Pd NPs being decorated over its surface. Pd NPs were immobilized following a post-functionalization of biguanidine over the core UiO-66-NH_2_ MOF than the typical surface deposition. The excellent chelating potential of biguanidine was exploited to deposit Pd NPs over it. Structural morphology and physicochemical features of the material were explored over different instrumental methods. Atomic mapping analysis displays the uniform dispersion of active sites throughout the surface matrix. The nanocatalyst has been deployed in the C–C coupling via Suzuki–Miyaura reactions under mild and green conditions to synthesize a wide variety of biphenyl derivatives affording outstanding yields. The robustness of the material has been validated by recycling it for 9 consecutive cycles without momentous loss of its reactivity. There is also negligible leaching of Pd species in the reaction medium, justifying its true heterogeneity.
